# **Tracking data of a Remotely Operated Vehicle and its tether using a motion capture system and a tension sensor**

**DOI:** 10.1038/s41597-025-06347-0

**Published:** 2025-12-03

**Authors:** Juri Khanmeh, Bilal Wehbe, Enrico Simetti, Giovanni Indiveri

**Affiliations:** 1 DIBRIS, University of Genoa, Via All’Opera Pia 13, 16145 Genoa, Italy; 2 RIC, German Research Center for Artificial Intelligence, Robert-Hooke Str. 1, 28359 Bremen, Germany; 3 Interuniversity Research Center on Integrated Systems for the Marine Environment, Via All’Opera Pia 13, 16145 Genoa, Italy

**Keywords:** Engineering, Physics

## Abstract

In this paper, we present a dataset related to the behaviour of underwater umbilicals of a Remotely Operated Vehicle (ROV), specifically the BlueROV2. The data were collected from three different sources: a Motion Capture System (mo-cap system), the onboard sensors of the BlueROV2, and a tension sensor. The mo-cap system tracks the motion of the ROV and its tether, while the tension sensor measures the force exerted by the cable tension on the surface side, near the tether drum. The dataset were acquired for research purposes, specifically for studying underwater cables in the context of modeling, estimation, and control using machine learning and data-driven control methods. They can serve as a benchmark for developing and validating underwater tether models, enabling researchers to develop control methods that consider tether drag or entanglement, improving navigation accuracy of ROVs, or developing automated tether management systems. This paper describes the experimental setup, data acquisition, and post-processing procedures. Furthermore, it provides illustrative plots to highlight key features of the dataset.

## Background & Summary

Remotely Operated Vehicles (ROVs) are among the most widely used underwater platforms for maritime inspection and operations, owing to their tether connection, which provides a continuous power supply and reliable communication with the offshore control station. At the same time, this physical connection is considered a drawback because the limited length of the cable restricts the vehicle’s motion. Moreover, ROV cables can easily become entangled with obstacles^1^, particularly since even small observation-class ROVs operate at depths of up to 300 m, requiring long tethers that increase the risk of entanglement. In such scenarios, the avoidance of cable entanglement is complicated by the increased cable length and the associated difficulty of controlling and estimating the cable shape^2^.

Previous studies have attempted to simplify the estimation of the ROV tether by adding ballasts and buoys^3^. However, in these works, the cable is represented using a simplified straight-line model, which corresponds to a specific case and does not capture the general behavior of an underwater cable. Additional research has investigated a vision-based cable shape identification method^4^. Alternative approaches have been studied, proposing active methods for cable control^5,6^. Both methods use a second vehicle to set a specific point of the cable in a known position. The first one uses an Autonomous Surface Vehicle (ASV) as the secondary vehicle. The controlled cable shape is based on the relative position between the ROV and the ASV. The second method utilizes another ROV and relies on the vision of the cable connecting both ROVs to estimate the position of the second ROV. Further studies have investigated the modeling of ROV cables^7,8^. In this context, the availability of ground-truth data plays a crucial role in supporting the validation of the developed models. For example, by conducting experiments in a water basin and recording the ROV position using a motion capture (mo-cap) system, the ROV localization system based on umbilical angle measurements has been tested and validated^9^.

However, when it comes to underwater cables, there is still a lack of ground-truth data regarding their shape and position. In other words, there are no reliable reference measurements against which the model outputs can be validated. For similar reasons, data-driven approaches to tether control have still been absent in this field. This lack of datasets stems from the difficulty in acquiring such information; in particular, the underwater localization data, as they require special maritime basins and underwater tracking devices. Indeed, cable modeling issues not only affect the ROV’s stability and the accurate reproduction of its nonlinear motion behavior^8^, but also limit the potential of one of the most promising configurations, the ASV-ROV combined system^10^. In such systems, the unknown shape and position of the tether hinder vehicle coordination and increase the risk of mobility restrictions due to cable entanglement with obstacles. Even if the tether management system (TMS) in an ASV-ROV configuration is equipped with a tension sensor to measure the cable tension, which can assist in estimating the tether length, the benefits of this sensor cannot be fully exploited without real data and an appropriate model of the tether behavior.

On these bases, we conducted several experiments using the BlueROV2 (an ROV commonly employed in research) and collected a dataset comprising various measurements from multiple sources. The dataset includes the ROV’s position and velocity, the positions of the markers placed along the tether, and MAVLink feedback messages containing sensor measurements such as attitude, battery status, raw and scaled IMU data, and internal and external pressure readings. The MAVLink messages also include command-related data, such as RC Input and RC Output. The RC Input consists of an array of control channels representing pilot commands (e.g., forward motion and yaw rotation), whereas the RC Output contains the pulse widths sent to individual servo output pins. This input-output data is particularly valuable for ROV simulation and control validation. Concerning the tether shape, the dataset contains the positions of each marker on the tether. The overall shape is estimated by interpolating the points (markers’ positions) with straight lines. Additionally, the dataset contains forces due to cable tension along a horizontal axis (along the pulley) and along the vertical axis.

A set of experiments was carried out, with the collected data summarized in Table 1. Two types of experiments were designed to address different scenarios, each supporting distinct modeling objectives. In the first type, a limited length of tether was released while keeping the cable’s surface attachment point fixed. Under these conditions, the cable closely followed the ROV’s motion. This configuration is particularly useful for studies aiming to simplify estimation by constraining cable dynamics, such as in ASV-ROV systems. In the second type of experiment, a longer length of tether was released, allowing the cable to move freely in the water with minimal influence from the ROV’s motion. This setup is better suited for research focused on modeling complex underwater cable dynamics, where fewer constraints are imposed on the cable’s shape and motion. In both experiments, the tether reel was not actively controlled: in the first case, it was locked, whereas in the second case, it was left free to unwind as required by the ROV’s motion. Since the tether is neutrally buoyant, the payout rate of the reel had a negligible effect on both the cable and the ROV’s dynamics.

**Table 1:** Overview of the data sources and associated measurements acquired during the experiments.

Data Source	Measured Data
ROV	Attitude, battery status, raw and scaled IMU data, pressure, RC Input, RC Output
Tension sensor	Horizontal and vertical forces
Mo-cap system	ROV position and velocity, tether shape

Each source provides complementary information, including ROV internal data, external tension measurements, and mo-cap tracking of the ROV and tether, enabling multi-sensor analyses.

The uploaded dataset comprises the complete set of experimental data collected simultaneously and is designed to support a range of applications by selecting the relevant data subsets. For example, investigations into the motion control of the ROV may utilize data such as the vehicle’s position, velocity, input commands, and thruster PWM signals. Alternatively, analyses focusing on the correlation between tether tension, cable length, and the ROV’s position and orientation can be conducted using the corresponding data. Additionally, the dataset includes recordings of the tether’s shape during ROV operation, making it suitable for studies on the dynamic behavior of underwater cables.

## Methods

**Fig. 1:**
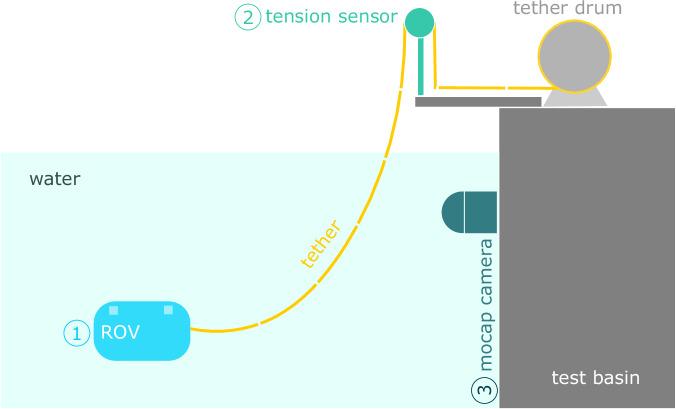
Experimental Setup: The ROV is deployed in the test basin and operated remotely, while the tension sensor measures the tether tension at the highest point outside the water, near the tether drum. The mo-cap system tracks the position of both the ROV and its tether. Data are acquired from three sources: the ROV, the tension sensor, and the mo-cap system.

Experiments were conducted by deploying an ROV in the test basin and remotely piloting it while collecting precise position data by a mo-cap system, as illustrated in Fig. 1. The mo-cap system tracks the motion of both the ROV and its tether. Additionally, a tension sensor was fixed on the surface near the tether drum to measure the tension applied to the tether. The tension is measured by letting the tether pass around a pulley mounted on the shaft of the tension sensor, after which it descends into the water to connect with the ROV. In parallel, commands and feedback messages transmitted via the MAVLink protocol from and to the ROV were logged. The following sections provide detailed descriptions of the data acquisition systems and methodologies employed.

### BlueROV2 Onboard Systems and Telemetry Logging

#### ROV

**Fig. 2:**
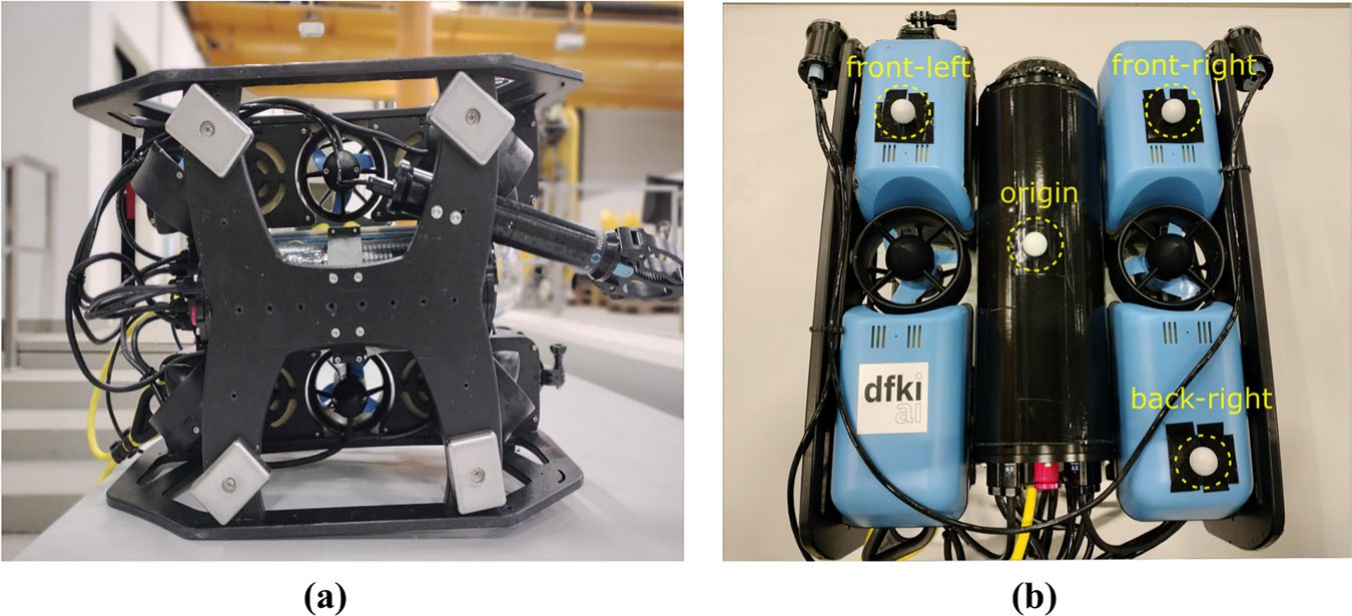
BlueROV2 Setup: **(a)** Bottom View: The position of the 4 ballasts for balancing the ROV and making it neutrally buoyant. **(b)** Upper View: Fixing 4 mo-cap markers to define a rigid body in the Qualisys Track Manager (QTM) software.

The core of the setup is the BlueROV2 (https://bluerobotics.com/store/rov/bluerov2/), a low-cost, highly modular remotely operated vehicle, powered by a lithium-ion battery and equipped with a Newton Subsea Gripper. The gripper is irrelevant to this experiment but influential in ballast placement as shown in Fig. 2a. The ROV was connected via a neutrally buoyant tether, specifically the Fathom ROV Tether, which consists of four twisted pairs and has a diameter of 7.6 mm, a minimum working bend diameter of 75 mm, a bending stiffness of 0.54N m^2^, and an axial stiffness of 4.3 × 10^4^ N. For more information regarding the physical specifications, the reader can refer to the tether cable’s technical details (https://bluerobotics.com/store/cables-connectors/cables/fathom-rov-tether-rov-ready/). As for the hydrodynamic coefficients of the ROV and the tether material properties, these were identified and published in^11,12^.

#### Sensors

The BlueROV2 is equipped with a range of onboard sensors, including a 6-DOF IMU, Dual 3-DOF magnetometers, an internal barometer, an external pressure/depth, a temperature sensor, and current and voltage sensors. The control architecture consists of an Autopilot, specifically the Navigator Flight Controller (https://bluerobotics.com/store/comm-control-power/control/navigator/) running the ArduSub (https://www.ardusub.com/) firmware, and a companion computer, running BlueOS (https://docs.bluerobotics.com/ardusub-zola/software/onboard/BlueOS-1.0/). The companion computer facilitates communication between the autopilot and the topside computer by relaying MAVLink messages over an Ethernet connection (https://ardupilot.org/dev/docs/mavlink-basics.html). For more information regarding the BlueROV2’s hardware connections, readers are encouraged to explore the provided link (https://www.ardusub.com/introduction/hardware-options/connection-diagrams.html).

#### ROS Topics

Although MAVLink messages can be monitored using the *MAVLink Inspector* tab in the QGroundControl interface, this tool does not support message logging. To address this limitation, we developed custom Robot Operating System (ROS) nodes that subscribe to MAVLink messages, convert them into ROS-compatible formats, and publish them on ROS topics. These topics are then recorded using the ROS2 bagging system, enabling time-synchronized logging of MAVLink data for offline analysis.

### ROV and Tether Pose Estimation via Optical Motion Capture System

#### Test Basin

**Fig. 3:**
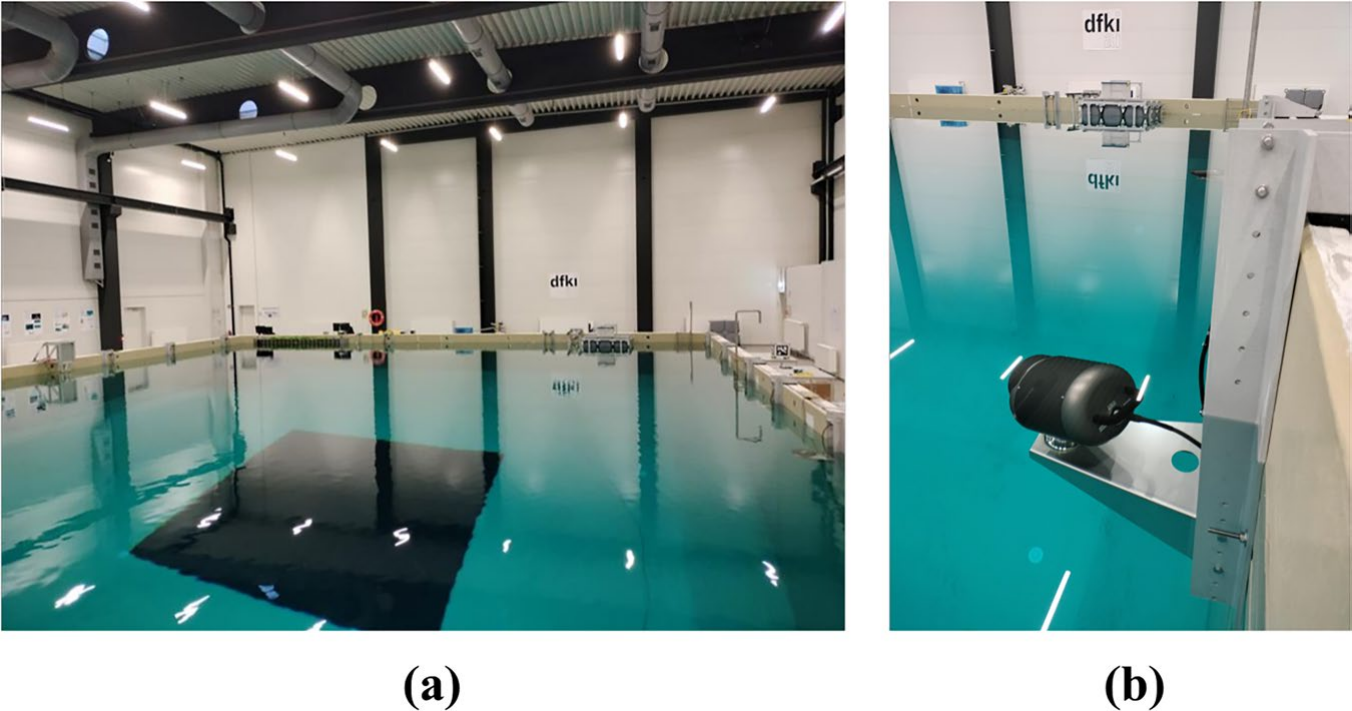
Mo-cap System Setup: **(a)** Test basin covered by 12 mo-cap cameras. **(b)** A mo-cap camera fixed inside the basin.

The experiments were conducted in the test basin of the Maritime Exploration Hall in the Robotics Innovation Center (RIC) in the German Research Center for Artificial Intelligence GmbH (DFKI). The basin measures 23 m × 19 m × 8 m filled with 3.4 million liters of saltwater. Precisely, the experiments were executed in the water having the following conditions: conductivity 2.06 ± 0.01 S/m, temperature 22.68 ± 0.01 °C, and pressure 103520.0 ± 0.3 Pa. Based on the Practical Salinity Scale of 1978 (PSS-78), the salinity and density of the water can be calculated. This facility provides controlled and observable conditions, enabling repeatable experiments regardless of external weather variations. The basin is equipped with an underwater motion tracking system for 3D motion capture. A total of twelve cameras detect optical markers on the robots and compute their precise position and orientations within the basin, as illustrated in Fig. 3a.

#### Motion Capture System

**Fig. 4:**
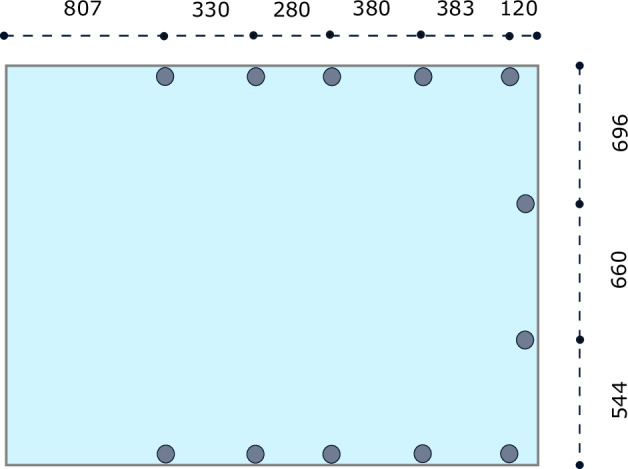
2D drawing of the upper view of the test basin: The gray dots are the mo-cap camera positions. (Units are in *cm*).

The mo-cap system installed in the basin of Maritime Exploration Hall at RIC has the following specifications: manufacturer: Qualisys AB; number of cameras: 12; resolution: 12 megapixels per camera; sampling rate: 250 Hz; accuracy: approximately 4 mm. The cameras are distributed along three edges of the basin near the surface of water, and tilted downward at an angle of approximately 10°, as shown in Fig. 3b. Five cameras are mounted on each of the long edges, and two cameras are installed on the short edge, as illustrated in Fig. 4. After calibrating each camera and adjusting *Exposure & Flash Time* and *Marker Threshold*, the system was configured with an *Exposure & Flash Time* of 500 us and a *Marker Threshold* of 20 %.

#### Markers

Two different types of markers were used for these experiments. The first type is super-spherical underwater mocap markers (https://www.qualisys.com/accessories/markers/super-spherical-markers/) of which four were mounted on the BlueROV2, as depicted in Fig. 2b. These markers were selected for their durability and reliability in prolonged underwater use, offering better performance than traditional taped mo-cap markers. In general, the markers are placed asymmetrically on the most visible surface of the vehicle, as seen by all cameras. In our case, since the 12 cameras are mounted near the top of the pool and tilted downwards, the upper surface of the ROV is the most visible. The specific marker positions were chosen to enable the identification of the ROV’s *body-frame coordinate system*. In particular

- The vector from the back-right marker to the front-right marker defines the x-axis direction.
- The vector from the front-left marker to the front-right marker defines the y-axis direction.
- An additional origin marker defines the origin of the body-frame.

**Fig. 5:**
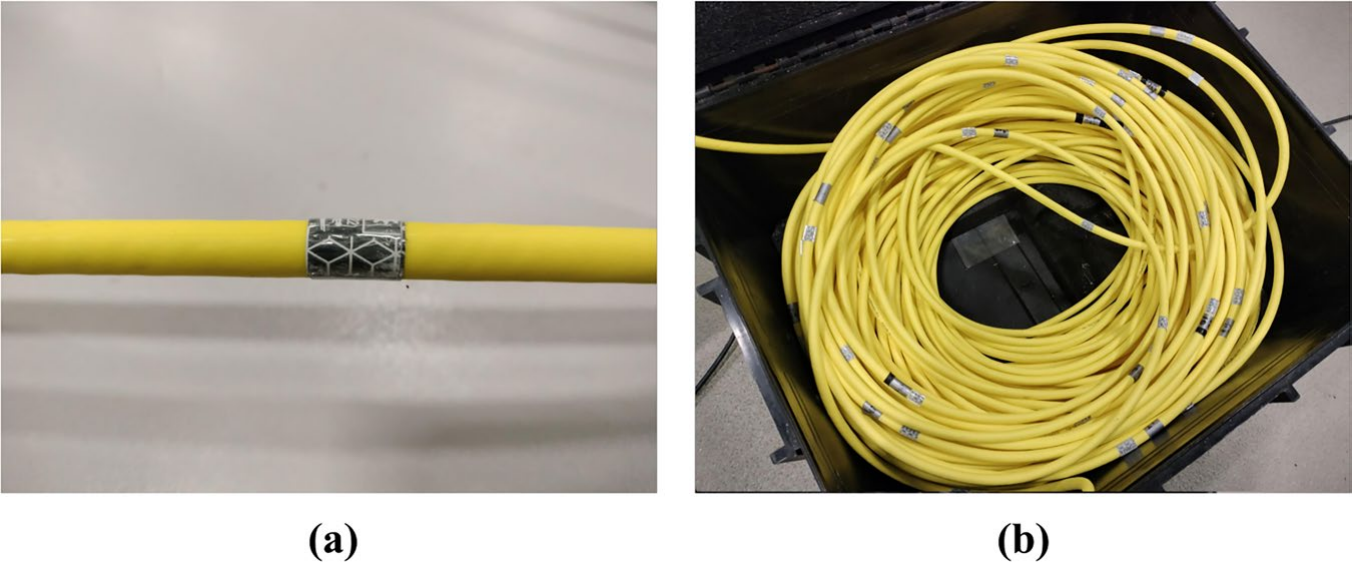
Tether Setup: **(a)** A single tape (marker) width [1.0 − 1.5] cm for the mo-cap system. **(b)** The entire marked tether.

The spherical markers were attached to the ROV using bolts fixed with strong waterproof tape (PATTEX Power Tape). Once the bolts were secured to the ROV surface, the markers were mounted by simply tightening them onto the bolts. The second type of used marker was the *retro-reflective underwater tape*. This tape was selected for marking the tether instead of using spherical markers, as it has minimal impact on the cable’s dynamical behavior. Strips of retro-reflective tape, each approximately 1.5 cm wide (Fig. 5a), were attached to the tether at regular intevals of 50 cm. Since the maximum cable length released during the experiments was about 13 m, only the first 20 m of the tether were marked, as shown in Fig. 5b.

#### Qualisys Track Manager (QTM)

QTM is the mo-cap software used for tracking object motion, post-processing, and data recording. It allows the definition of rigid bodies, enabling the BlueROV2 to be defined and tracked in real time. Consequently, its position and velocity were computed and published in real time as ROS topics. However, the tether is not modeled as a rigid body due to its flexible nature and complex behavior. Since no predefined model was available (and generating one was not feasible), tether tracking data had to be manually labeled during the post-processing phase. This process is discussed in more detail in the upcoming section *post-processing*.

### Tether Tension Sensing Setup

#### Tension sensor

**Fig. 6:**
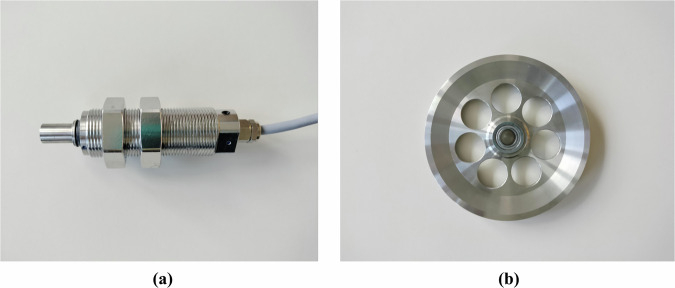
Tension Sensor Components: **(a)** Sensor RFS® 150 XY. **(b)** Pulley.

For collecting the tension data, the radial force sensor RFS® 150 XY (https://honigmann.com/k7/g75/i456/Tension-sensors-ALL-2-axis-radial-force-sensor-RFS150-XY.html) is utilized. The sensor features two orthogonal measurement axes, with a splash-proof surface for wet or very dusty environments (Fig. 6a). This specific tension sensor allows us to estimate the resultant tension force vector without requiring knowledge of the exact wrapping angle by combining two radial components. The sensor was calibrated using precise weights, and the orthogonal axis was precisely aligned to the vertical and the horizontal axes.

#### Pulley

A lightweight stainless-steel U-groove pulley was used in conjunction with the sensor, specifically designed for compatibility with the RFS® 150 XY. The pulley features a low-mass construction with lightening holes and has an outer diameter of 108 mm, a root diameter of 95 mm, and a groove width of 12 mm. It is optimized for cables with a radius of 4 mm and has a bearing journal of diameter 10 mm (see Fig. 6b).

#### Platform

**Fig. 7:**
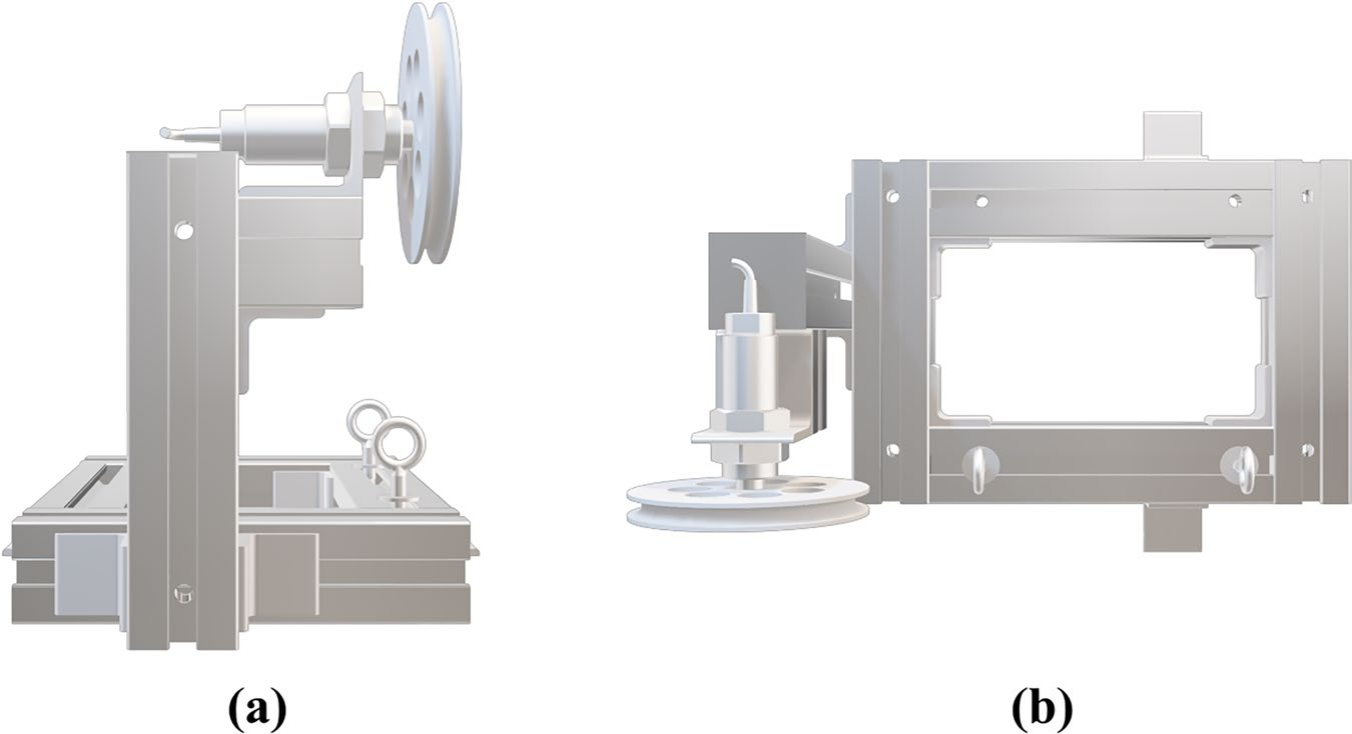
3D model of the assembly of the platform and the tension sensor. The assembly is made from: a tension sensor, a pulley, a platform made by aluminum frames, and two hooks for ensuring the cable aligns to the vertical plane of the pulley. **(a)** Side view. **(b)** Upper view.

The sensor and pulley assembly is mounted on a custom platform constructed employing 45 mm ×45 mm aluminum profiles, as shown in Fig. 7. To ensure that the tether enters the pulley groove in a consistent plane, two guiding hooks were installed upstream, aligned with the pulley groove. This prevents the tether from slipping off during cable deployment and maintains a controlled wrapping geometry. The sensor is positioned slightly higher than the hooks to guarantee a minimum wrapping angle around the pulley, improving the consistency of the measurement.

#### Conditioning circuits

Signal conditioning was performed using the Tensiotron® TS 621, which processes the raw signals from the sensor and outputs the amplified and filtered data. For more information about the board, the reader can refer to the link (https://www.honigmann.com/page.php?m_pro=14&lang=2).

#### Acquisition software

HCC-Easy software (https://www.honigmann.com/page.php?m_pro=401&lang=2) was used for data acquisition and logging. The program provides an integrated platform for real-time data acquisition, recording, and analysis of tension data.

### Data Acquisition

**Fig. 8:**
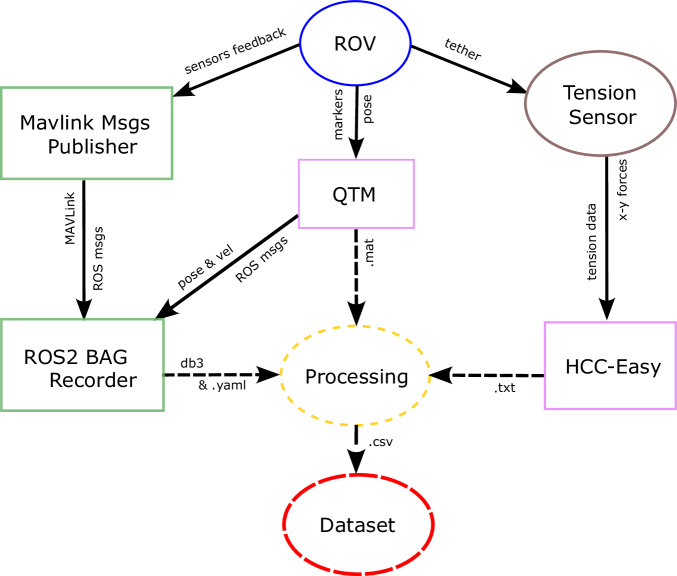
Software architecture of the data acquisition system. Color legend: *blue* represents the robot, *pink* represents software, *brown* represents sensors, *green* represents the developed packages, *yellow* represents the post-processing stage, and *red* represents the final dataset. The labels near the dashed arrows indicate the corresponding data formats.

All data were acquired simultaneously. The final architecture of the data acquisition system is illustrated in Fig. 8. To save the MAVLink messages sent from the onboard computer in the ROV, we developed a ROS package containing a node that connects to the MAVLink UDP port, receives the MAVLink messages, converts them to time-stamped ROS messages, and publishes them on ROS topics. In the meantime, the QTM receives the image frames from the mo-cap cameras, computes the positions and velocities of the ROV, and publishes them in ROS topics. The *ROS2 Bag Recorder* node subscribes to these topics and stores the messages in a ROS bag. Tracking data not detected in real time, such as the tether’s markers, are labeled manually after recording using QTM and exported following the post-processing stage. Meanwhile, HCC-Easy handles the tension data received from the tension sensor and records it as a .HED file. Data acquisition was performed using three different computers: the first, connected to the mo-cap system, runs QTM; the second, connected to the BlueROV2, runs the ROS nodes; and the third, connected to the tension sensor, runs HCC-Easy. All three computers were connected to the same local network to ensure synchronized clocks.

### Post-Processing

In general, we processed the data as follows.

**Fig. 9:**
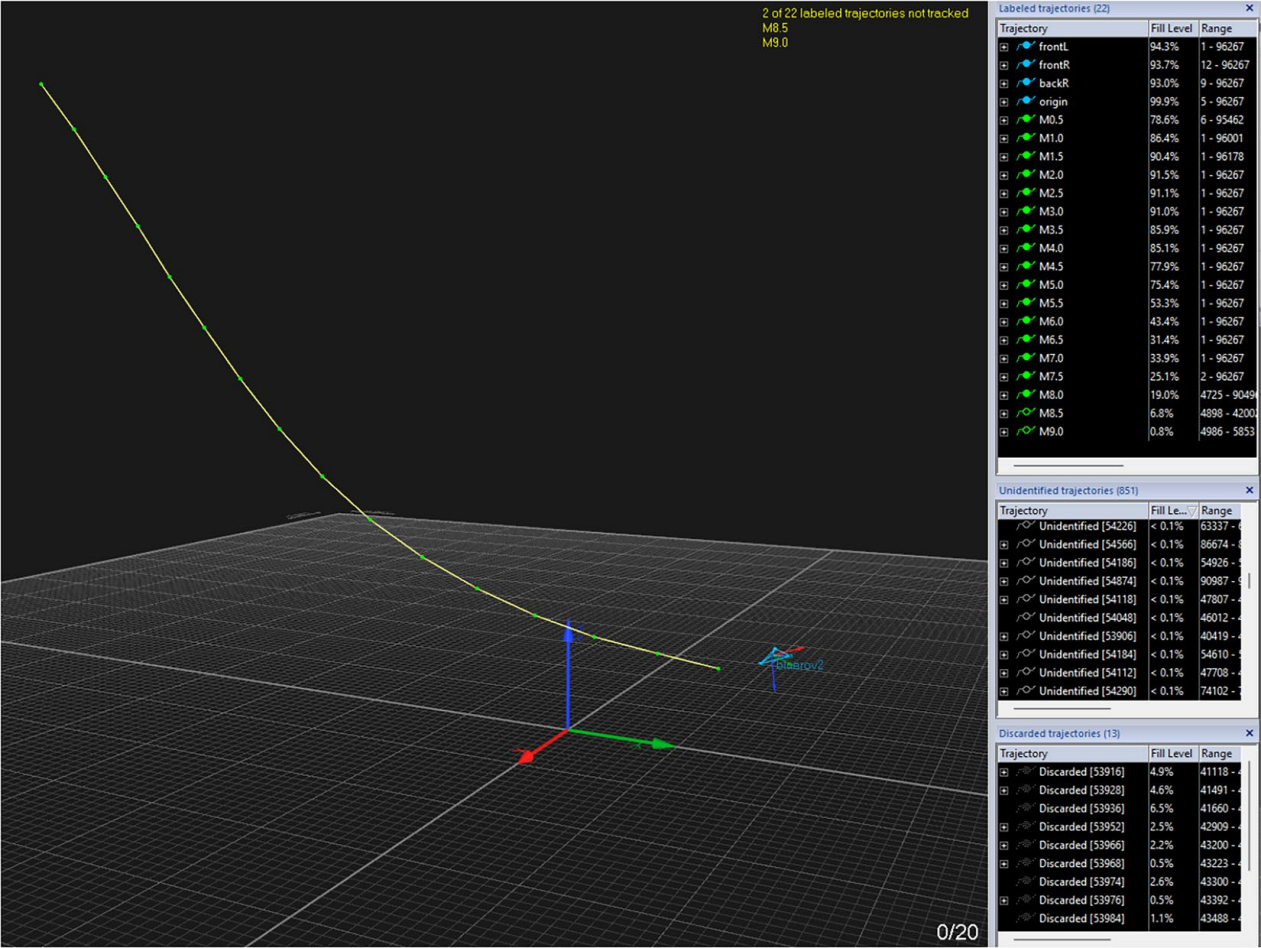
Manual annotation of the markers using QTM: Each marker of the tether had to be annotated since there was no automatic labelling due to the lack of the tether model. The *blue* lines represent the rigid body of ROV, and the *green* dots are the markers of the tether, while the *yellow* lines interpolate the tether markers with straight lines to represent the cable. In the upright panel *Labelled trajectories*, the labelled markers are listed; the *blue* ones are the ROV markers, and the *green* ones are for the tether. The last two markers (M8.5 and M9.0) are not filled because they are not detected by the cameras at that moment. The unlabelled and discarded trajectories are listed below in *Undefined trajectories* and *Discarded trajectories*.
- Gap-filling and Smoothing Tracking Data: QTM offers the capability to process the recorded data, like smoothing the trajectories by removing spikes. In contrast, when the markers are not detected, there are gaps in the trajectory. In such a case, we needed to use gap-filling functions to export continuous trajectories.
- Filtering Tension Signal: HCC-Easy provides a signal filtering function to remove noise and spikes. However, the recorded data was relatively clear and acceptable, so the tension data presented in this paper is the raw data without applying any additional digital filters.
- Data Synchronization: Since the data is acquired from three different independent sources, one of the challenging parts in acquiring the dataset was the time synchronization. The ROS messages stored from the ROS topics are all time-stamped. The data exported from QTM has the time stamp of the first frame and the rate of the stored frames. Regarding the tension measurements, the data were time-stamped starting from zero. By knowing the time of file creation, we managed to shift the time and obtain the correct time stamp for each frame. To get the time-stamp of the file creation, we used FileTime MATLAB toolbox^13^ (https://www.mathworks.com/matlabcentral/fileexchange/24671-filetime).
- ROV Orientation Correction: Both the IMU and the mo-cap system calculate the orientation of the ROV in real time. The IMU estimates it using an EKF, while the mo-cap system determines it by detecting the markers that define the rigid body. In Fig. 10, the ROV’s orientation angles from both sources are plotted. However, since the reference frames were not initially unified, the two representations differ. Specifically, the IMU provides the ROV’s orientation in roll-pitch-yaw (RPY) form with respect to an inertial frame whose axes are aligned with the North-East-Down (NED) frame. In contrast, the mo-cap system outputs the orientation of the ROV frame with respect to its own local base frame. Consequently, the RPY angles from the two systems are not directly comparable. To align the data, a rotation matrix is applied to transform the mo-cap orientation from its local frame to the NED frame, as shown in Fig. 11. After this correction, both RPY representations refer to the same reference frame and can be meaningfully compared. In general, the orientation from the mo-cap system is relatively accurate especially for the yaw angle; however, there are some discontinuities especially on the roll angle, due to the missing detection of some markers at certain time intervals as shown in Fig. 11, around the time interval [25, 75] s and [160, 180] s, where there are sudden flips on the roll angle. On the other hand, the IMU always gives continuous and precise angle estimation for the roll and the pitch; however, the yaw angle drifts over time, especially when the ROV approaches metal constructions due to its effect on the magnetometer. Additionally, there is the issue discussed in the Blue Robotics forum (https://discuss.bluerobotics.com/t/yaw-drift-due-to-increase-of-ekf-gyro-bias-estimation/5490), where the reason of this drift is assumed to be due to the Gyro-Z bias estimations in EKF that is drifting over time. To avoid any kind of confusion in the dataset, the final ROV’s orientation data contains the roll and pitch from the IMU and the shifted yaw to align with the one from the mo-cap system.

- Labeling Mo-cap Markers: QTM can track the trajectory of rigid bodies if their markers are defined as a rigid body in the system before recording. In this way, it can detect the rigid body in real time and publish its position and velocity on ROS topics during the experiment. However, the tether’s markers are not defined as a rigid body because the distances between markers change according to the tether’s shape. Moreover, since the underwater cable model is quite complex, the tether cannot be detected automatically in real time. For these reasons, the trajectories of the tether’s markers were labeled manually after the experiment. Manual labeling refers to assigning names to the trajectories detected by the mo-cap system. A screenshot of the QTM interface is shown in Fig. 9. On the left side, the main window shows the detected trajectories by the mo-cap: the ROV rigid body is shown in blue, and the tether markers are depicted in green. The yellow lines connect the tether markers for visualizing the tether. On the top-right, the list of *Labelled trajectories* is shown. The blue ones, which are the trajectories of the ROV markers, are detected automatically. On the other hand, the green ones, which are the trajectories of tether markers, are labelled manually. Initially, all trajectories (except those of rigid bodies) are marked as *Undefined trajectories*. By clicking on a single trajectory (represented by a dot in QTM) and assigning a label to it, the trajectory becomes a labelled one. This process is repeated for all the markers. The tether markers were labeled using the following naming ‘MX’, where ‘M’ stands for ‘marker’ and ‘X’ is substituted by the length from the starting point of the cable from the ROV side. For example, the first five markers of the tether are named M0.5, M1.0, M1.5, M2.0, M2.5, and so on. In the middle-right panel of QTM, there is a list of *Undefined trajectories*. Due to light reflections and other disturbances affecting the mo-cap cameras, QTM sometimes detects incorrect trajectories, which are initially listed under *Undefined trajectories*. By filtering these trajectories, incorrect and unnecessary ones can be discarded and moved to the *Discarded trajectories* list in the bottom-right panel (see Fig. 9).

**Fig. 10:**
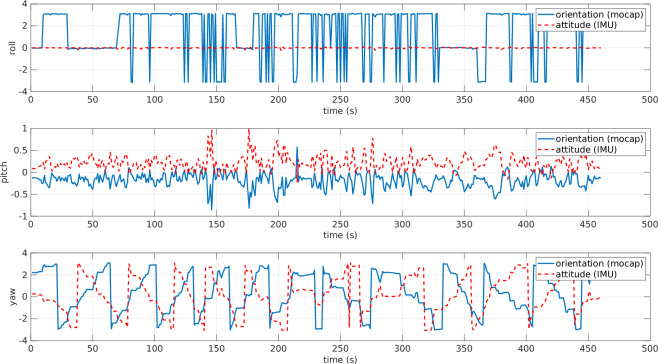
ROV orientation angles: raw data from mo-cap and IMU are plotted on different reference frames. The angles are in radians. The data from the mo-cap is projected on mo-cap reference frame, while data from IMU sensor is projected on the NED frame. Hence, the data are completely misaligned.

**Fig. 11:**
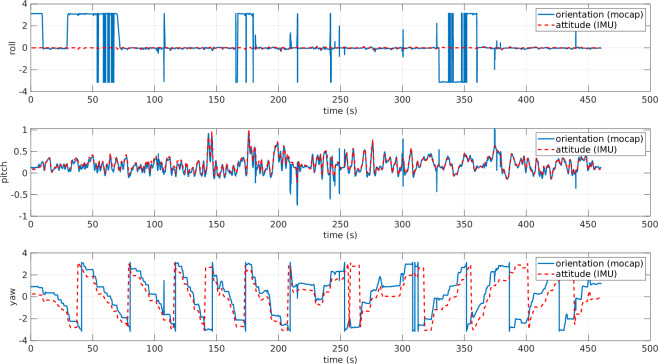
ROV orientation angles: data from the mo-cap and IMU projected on the same NED frame. The angles are in radians. The two plots from roll and pitch are aligned after the first step of post-processing. On the other hand, there is still a drift on the yaw data.
- Calculating Tension Force: The tension sensor measures the forces *F*_*x*_ and *F*_*y*_ generated by the tether on the pulley (Fig. 12), which is not equal to the tension force. In this section, we explain how to calculate the tether tension using the annotation of angles and arms as illustrated in Fig. 12. According to the datasheet of the tension sensor, the resultant force *F*_*R*_ is calculated based on the tension force *F*_*z*_ and the wrapping angle *α*:
  1 $${F}_{R}=2{F}_{z}\sin \left(\frac{\alpha }{2}\right)=\sqrt{{F}_{x}^{2}+{F}_{y}^{2}},$$
  where *F*_*x*_ and *F*_*y*_ are the measured forces by the sensor on the vertical and horizontal axes, respectively. In our application, the wrapping angle is not fixed since the tether follows the motion of the ROV. Hence, to calculate the wrapping angle *α*, the angles *φ*, *ϵ* and *β*, as illustrated in Fig. 12, should be calculated using the following equations:
  2 $$\varphi ={\tan }^{-1}\left(\frac{{F}_{x}}{{F}_{y}}\right);$$
  3 $$\epsilon ={\cos }^{-1}\left(\frac{C}{\sqrt{{A}^{2}+{B}^{2}}}\right)-\beta ;$$
  4 $$\beta ={\tan }^{-1}\left(\frac{A}{B}\right);$$
  5 $$\frac{\alpha }{2}+\epsilon =\varphi .$$
  By substituting equations (2), (3), and (4) into (5), the wrapping angle *α* can be expressed as
  6 $$\alpha =2(\varphi -\epsilon )=2\left[{\tan }^{-1}\left(\frac{{F}_{x}}{{F}_{y}}\right)+{\tan }^{-1}\left(\frac{A}{B}\right)-{\cos }^{-1}\left(\frac{C}{\sqrt{{A}^{2}+{B}^{2}}}\right)\right],$$
  where *A*, *B*, and *C* are known geometrical parameters with values *A* = 285 mm, *B* = 160 mm, and *C* = 54 mm. Once the wrapping angle *α* and the resultant force *F*_*R*_ are determined, the tension force *F*_*z*_ is calculated as
  7 $${F}_{z}=\frac{\sqrt{{F}_{x}^{2}+{F}_{y}^{2}}}{2\sin (\alpha /2)}.$$

**Fig. 12:**
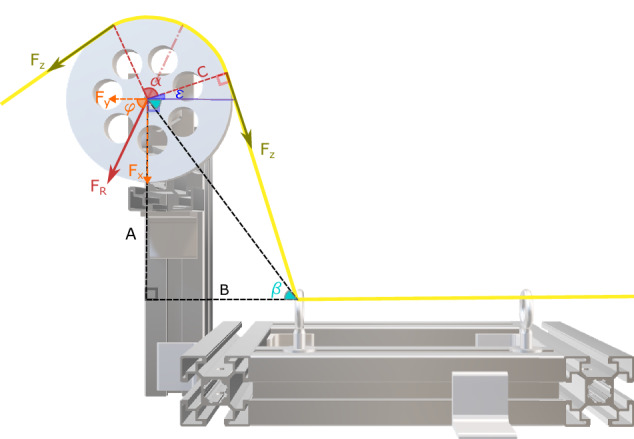
Tether Tension calculation: *F*_*z*_ is the tension force of the cable, *F*_*R*_ is the resultant force, *F*_*X*_ and *F*_*Y*_ are the projections of the resultant force on the vertical and horizontal axes, respectively, *α* is the wrapping angle, and *C* is the radius of the pulley. *A* and *B* are the vertical and the horizontal distances between the center of the pulley and the center of the left hook.
- Exporting Data: QTM can export data in several formats, including C3D, TSV, AVI, FBX, or MATLAB files. In this work, mo-cap data were exported in MATLAB file format. MAVLink messages were saved using a ROS2 bag recorder (https://docs.ros.org/en/humble/Tutorials/Beginner-CLI-Tools/Recording-And-Playing-Back-Data/Recording-And-Playing-Back-Data.html), producing *metadata.yaml* and DB3 files. Tension sensor data were exported as .txt files by HCC-Easy. To unify the data for analysis, all data were processed in MATLAB and converted to Comma-Separated Values (.csv) format after synchronization. The final curated dataset is therefore provided entirely in .csv format.

## Data Records

**Fig. 13:**
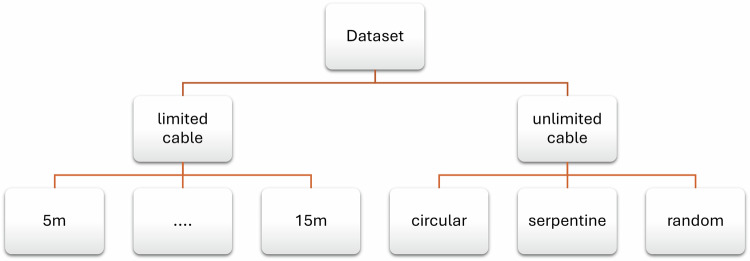
Structure of the dataset folder and its subfolders.

The dataset is available on Zenodo^14^ 10.5281/zenodo.17360027. The dataset is organized according to different experiments as shown in Fig. 13. These experiments can be classified into two main categories: those conducted with a limited tether and those conducted with an unlimited tether. In the former case, the tether length was varied from 5 m to 15 m. In the latter, three experiments were executed: serpentine path, random path, and circular path. Each folder (experiment) contains the following data file:

1. MAVLink messages from the ROV:
  Attitude
  Battery Status
  IMU RAW
  IMU SCALED
  RC IN
  RC OUT
  Pressure

2. Tracking data from the mo-cap system:
  ROV’s position (x,y,z)
  ROV’s orientation (x,y,z,w)
  ROV’s velocity (linear and angular)
  Tether’s shape; which is represented by the tether’s markers positions (x,y,z)

3. Tension measurements from the tension sensor
  Horizontal force of the tether
  Vertical force of the tether

The whole dataset is in Comma-Separated Values (.csv) format, and the first column of each file is the time.

## Technical Validation

**Fig. 14:**
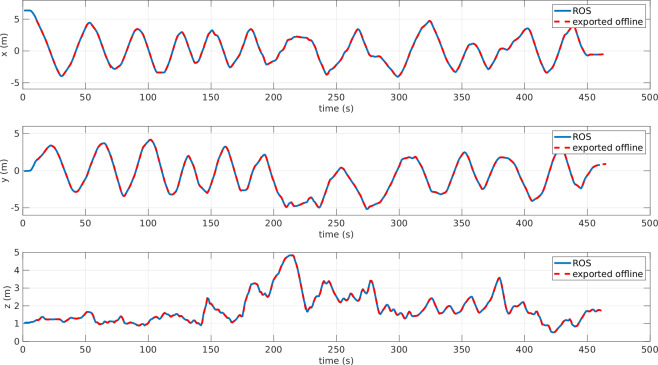
ROV’s cartesian position [x,y,z] - experiment: circular path. The *blue* line is the position of the rigid body and published in real-time in ROS topic. The *red* dashed line is the position of the marker of the origin of ROV exported by QTM after the recording.

In this section, we present several plots generated by a MATLAB script to visualize and verify the quality of the acquired dataset. Since the data were collected from three different sources and computers, the first step was to verify the synchronization of the data after post-processing. In Fig. 14, we plot both the ROV position acquired from the ROS topics, which was recorded online in ROS Bag, and the ROV marker’s position exported offline using QTM after the recording. This comparison verifies the synchronization between the ROS bag data and the QTM data.

**Fig. 15:**
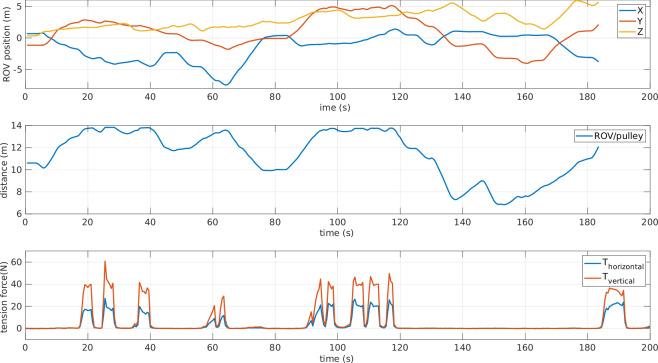
Tension vs. Distance - experiment: random motion with fixed tether length (14m).

To verify the synchronization with the tension data, the ROV position and the tension measurements were plotted together, as shown in Fig. 15. Knowing the position of the pulley with respect to the mo-cap system frame (*x* = 3.299 m, *y* = −8.510 m, *z* = 8.459 m), the distance between the ROV and the pulley was computed and plotted, as illustrated in Fig. 15. In this experiment, the maximum tether length was fixed at 14 m; consequently, when the distance between the pulley and the ROV approaches 14 m, the tension increases. The plot in Fig. 15 spans a relatively large timescale of 200 s. However, the data were also examined at smaller timescales, confirming that the signals are well synchronized.

**Fig. 16:**
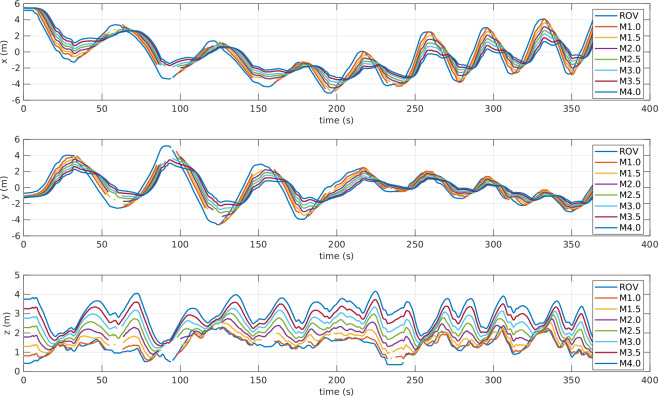
Markers’ Position [x,y,z] - experiment: circle path. In the legend of the plot, MX.0 stands for the marker on the tether at *X* m far from the ROV.

After verifying the synchronization of the entire dataset, various plots were generated. Starting with the tether’s markers, Fig. 16 shows the Cartesian positions of the ROV origin marker along with the first four tether markers. When the tether is vertical, the distance between successive markers is nearly constant. This is clearly visible in the third subplot (z-axis) of Fig. 16. The discontinuous trajectories are caused by temporary loss of marker detection by the mo-cap cameras during those intervals.

**Fig. 17:**
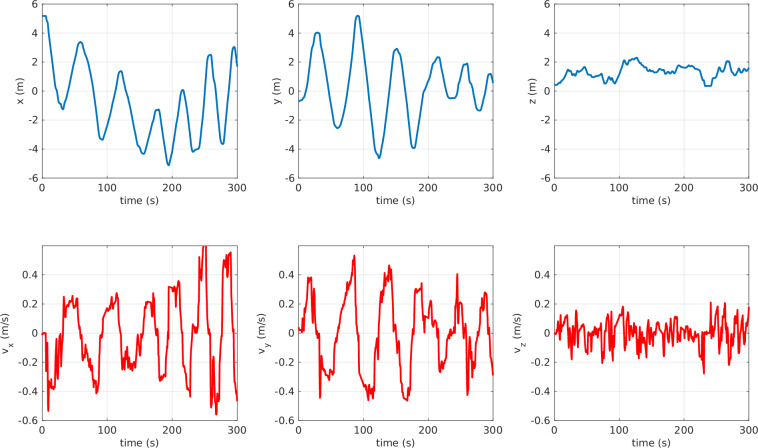
Rigid body tracking data: ROV’s position and velocity. The *blue* lines represent the ROV’s position, and the *red* lines represent its velocities.

A sample of the ROV’s position and velocity data is presented in Fig. 17. These measurements are obtained online from QTM, which detects the rigid body and publishes its position and velocity to the corresponding ROS topics, as illustrated in the data acquisition diagram shown in Fig. 8.

**Fig. 18:**
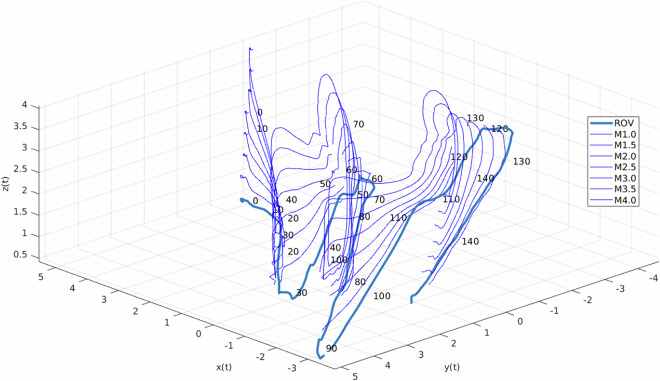
Time-labeled 3D trajectory of the markers. Experiment: serpentine path. To provide additional context, time labels are placed along the 3D trajectory at 10 s intervals. The thick *blue* line represents the ROV’s trajectory.

Another interesting plot is the 3D track of the markers, reported in Fig. 18. In this plot, the 3D trajectory of each marker is illustrated. Consequently, the continuity of each marker’s trajectory was validated.

**Fig. 19:**
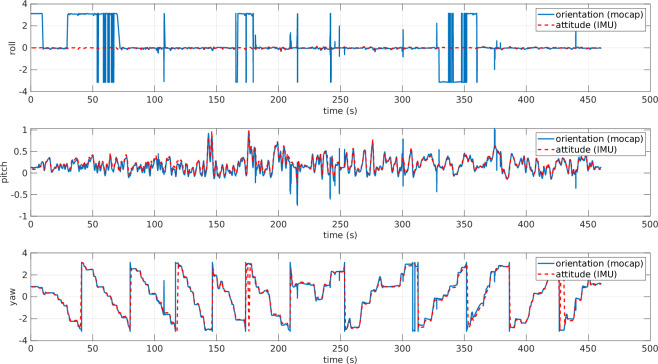
ROV orientation angles after post-processing (shifted yaw). The angles are expressed in radians. The three orientation components (roll, pitch, and yaw) are aligned.

**Fig. 20:**
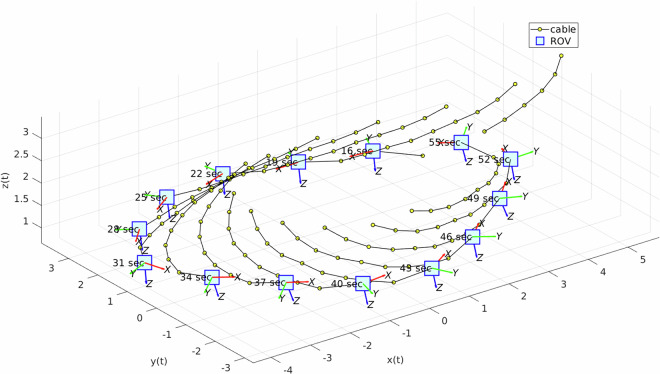
Time-labeled 3D view of the ROV and its tether (time interval: 3 s).

Another important aspect to consider is the orientation of the ROV. After applying the yaw angle shift discussed in the post-processing section, the resulting plots were generated, as shown in Fig. 19, where the three angles are aligned. To avoid confusion in the dataset, only the final version of the orientation (obtained from the IMU after shifting the yaw to align with the mo-cap system) was uploaded. Once the ROV orientation was corrected, we plotted the 3D position and orientation of the ROV along with its tether, as shown in Fig. 20. The ROV’s body frame in Fig. 20 was plotted using the plotframe MATLAB toolbox^15^ (https://www.mathworks.com/matlabcentral/fileexchange/156419-plotframe-plot-a-3-d-cartesian-coordinate-system?s_tid=srchtitle.

**Fig. 21:**
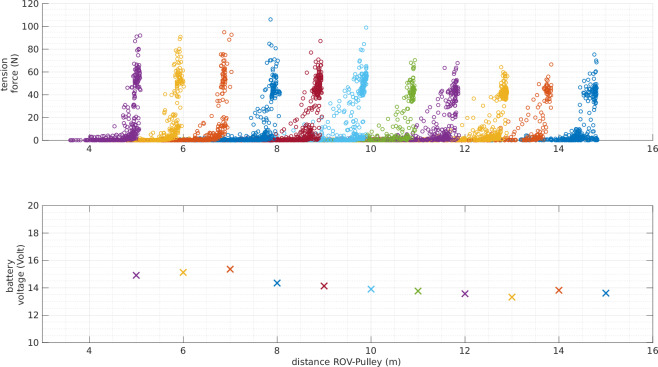
Tension force, ROV battery voltage, and the distance between the ROV and the pulley. The plot represents eleven experiments, in each experiment the tether length is fixed to a specific length (5, 6, 7, .., 15) m. Each color in the plot represents one experiment, for instance, in the *red* plot, the tether is fixed at 9 m since the maximum length is 9 m.

The final plot represents the tension force retrieved from the tension sensor, the battery voltage from the BlueROV2, and the distance between the ROV and the pulley of the tension. The correlation between the drop of the battery voltage and the maximum tension force is remarkable, as shown in Fig. 21.

After presenting the dataset and its main components, it is also important to discuss how these data can be used. The dataset provides a comprehensive benchmark of synchronized measurements that can be selectively exploited depending on the specific application. For instance, in an ASV-ROV cooperative vehicle system, the TMS must release an appropriate length of cable: if the cable is too short, it restricts the ROV’s motion, whereas if it is too long, it increases hydrodynamic drag. In other words, the reference cable length needs to be computed in real time to optimize the power consumption of the coupled vehicles.

**Fig. 22:**
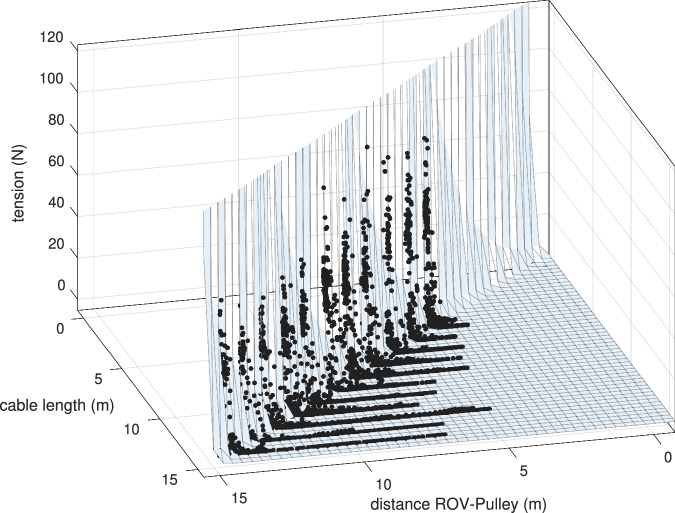
Curve fitting: a 3D plot was generated for following data: tension, cable length and the distance between the ROV and the tension sensor pulley. The *black* dots are the acquired data, and the *light blue* mesh is the fitted surface.

**Fig. 23:**
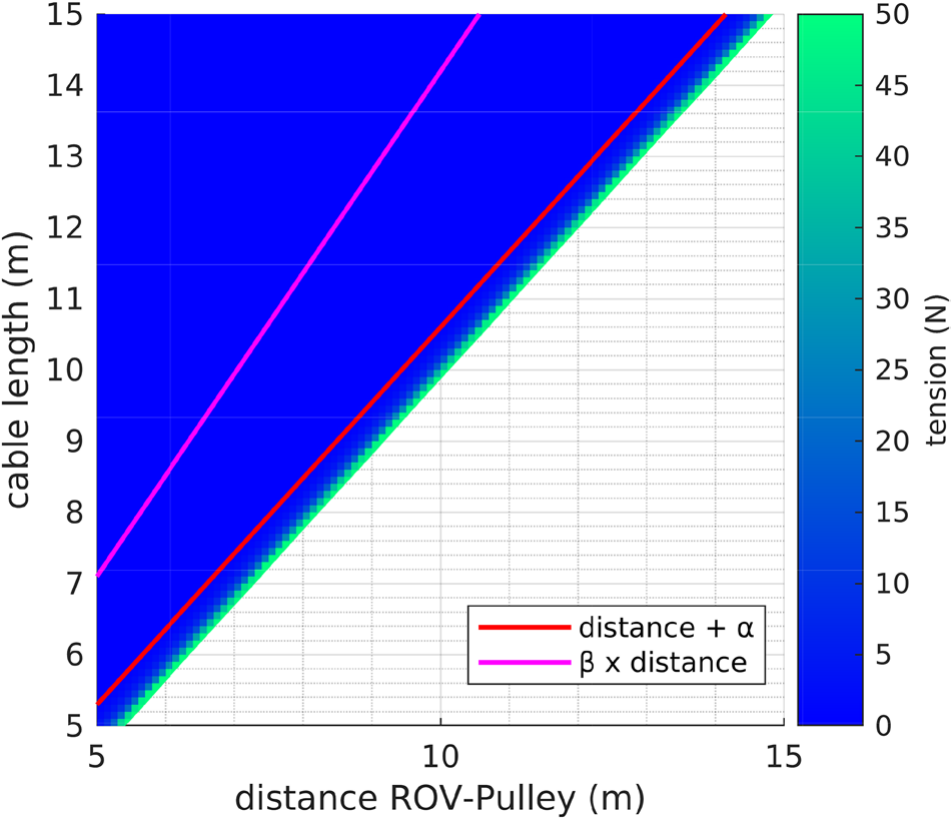
Heat map for tension model: After deriving the model of the BlueROV2 tether, the final surface equation (8) is plotted in this heat map. The area between the two lines is defined as the accepted region for the cable length. *α* and *β* are two positive parameters for setting the accepted region.

As an example of such potential use, the dataset was exploited to derive a tether model based on the measured tension, the distance between the ROV and the pulley, and the length of the released tether. A three-dimensional model was obtained through surface curve fitting, as shown in Fig. 22. The resulting surface fit equation is given by

8 $$f(x,y)=a\,\exp (bx+cy)$$

where *a*, *b*, and *c* are fitting parameters. *x* is the cable length, *y* is the distance between the ROV and the pulley, and *f* represents the cable tension. The ranges of the coefficients and the corresponding goodness-of-fit metrics are summarized in Tables 2 and 3, respectively. The fitted surface represented in equation (8) is also represented as a 2D heatmap in Fig. 23. This model defines an accepted region that is bounded between two curves. The accepted region corresponds to the admissible cable lengths given the distance between the ROV and the pulley. Ensuring that the cable length remains within this region guarantees that the tether is not excessively long, since maximum bounds are imposed by the upper limit (pink line in Fig. 23, while being sufficiently longer than the distance between the ROV the ROV’s motion (red line in Fig. 23).

**Table 2:** Fitted coefficients *a*, *b*, and *c* of the exponential surface model relating the ROV distance from the pulley, tether length, and tension.

Coefficient	Value	Lower Bound	Upper Bound
*a*	0.4682	0.4724	0.4766
*b*	7.6912	7.6701	7.7123
*c*	−7.2578	−7.2784	−7.2372

The table also reports the corresponding lower and upper bounds.

**Table 3:** Goodness-of-fit statistics for the exponential surface model relating the ROV distance from the pulley, tether length, and tension.

Statistical Measure	Value
Confidence bounds (CB)	95%
Sum of Squared Errors (SSE)	2.3351 × 10^3^
Coefficient of Determination (*R*^2^)	0.9989
Adjusted Coefficient of Determination (Adjusted *R*^2^)	0.9989
Root Mean Square Error (RMSE)	0.4835

**Table 4:** Summary of dataset files containing onboard sensor and system measurements.

File	Variable	Unit
attitude.csv	roll, pitch, yaw	rad
	roll_speed, pitch_speed, yaw_speed	rad/s
battery.csv	id	—
	voltages_01	V
imu_raw.csv	time_usec	*μ*s
	xacc, yacc, zacc	—
	xgyro, ygyro, zgyro	—
	xmag, ymag, zmag	—
imu_scaled.csv	time_usec	*μ*s
	xacc, yacc, zacc	G
	xgyro, ygyro, zgyro	rad/s
	xmag, ymag, zmag	mgauss
pressure/pressure2.csv	time_boot_ms	ms
	press_abs	Pa
	temperature	°C
rc_in.csv	rssi, channel_x (*x* ∈ [1, 18])	—
rc_out.csv	rssi, channel_x (*x* ∈ [1, 16])	—

Each file corresponds to a MAVLink message type and includes variables with their respective physical units.

**Table 5:** Summary of dataset files containing mo-cap measurements and post-processed quantities.

File	Variable	Unit
tension.csv	Tension_horizontal_Fy, Tension_vertical_Fx	N
pose.csv	pos_x, pos_y, pos_z	m
velocity.csv	linear_x, linear_y, linear_z	m/s
	angular_x, angular_y, angular_z	rad/s
qtm_rov.csv	rov_front_left_x, rov_front_left_y, rov_front_left_z,	m
	rov_front_right_x, rov_front_right_y, rov_front_right_z,	m
	rov_origin_x, rov_origin_y, rov_origin_z,	m
	rov_back_right_x, rov_back_right_y, rov_back_right_z	m
qtm_tether.csv	MX_x, MX_y, MX_z, *X* ∈ [0.5, 1.0, 1.5, . . . ]	m
	(X: distance in decimeters from the starting point of the cable)	

This data collection includes ROV pose, velocity, tension data, and 3D marker trajectories from the Qualisys tracking system.

## Data Availability

The dataset is available on Zenodo^14^ 10.5281/zenodo.17360027. The dataset is organized according to different experiments as shown in Fig. 13. These experiments can be classified into two main categories: those conducted with a limited tether and those conducted with an unlimited tether. In the former case, the tether length was varied from 5 m to 15 m. In the latter, three experiments were executed: serpentine path, random path, and circular path. The final dataset files are in Comma-Separated Values (.csv) formats, and summarized in Tables 4 and 5. The first column of all the files is the *time* in seconds.

## Code availability

The custom MATLAB scripts for post-processing the data are available on Zenodo^14^ 10.5281/zenodo.15976877under the *MATLAB codes* folder. Post-processing here refers to converting the dataset into .csv files and correcting the ROV’s orientation, as illustrated in Fig. 8. Regarding the software versions used in the experiments, QTM was version 2024.2, HCC-Easy was version 5.01.08, and ROS was ROS2 Humble.
